# The internal mammary artery perforator flap for neck reconstruction after palliative resection of advanced anaplastic thyroid cancer: a case report

**DOI:** 10.1186/s13256-022-03712-0

**Published:** 2023-01-09

**Authors:** Vera Amrillaeva, Henning Dralle, Frank Weber, Frauke Deneken, Farhad Farzaliyev

**Affiliations:** 1 Division of Endocrine Surgery, Department of General, Visceral and Transplantation Surgery, University Hospital Essen, University Essen-Duisburg, Essen, Deutschland; 2 Department of Plastic, Reconstructive and Aesthetic Surgery, University Hospital Essen, University Essen-Duisburg, Essen, Germany; 3 Department of Hand-, Plastic, Reconstructive and Burn Surgery, BG Klinik, Eberhard Karls University Tuebingen, Schnarrenbergstraße 95, 72076 Tübingen, Germany

**Keywords:** Advanced anaplastic thyroid cancer, Palliative resection, Internal mammary artery perforator flap

## Abstract

**Introduction:**

Defects of the neck after palliative resection of exulcerated tumors could be reconstructed with different skin flaps.

**Case presentation:**

The present report describes the case of a 40-year-old Caucasian female patient with advanced anaplastic thyroid cancer. The exophytically growing, bad-smelling massive exulcerated tumor caused an esthetic defect, neck mobility restrictions, and mental state deterioration.

**Primary diagnosis, interventions, and outcomes:**

Palliative debulking of the tumor was performed. The 10 × 5 cm skin defect of the neck was successfully reconstructed with an internal mammary artery perforator island flap. The donor site was closed primarily. The patient had an uneventful clinical course; the cosmetic results and mental state were very pleasing.

**Conclusions:**

The present case illustrates that palliative resection of the tumor and plastic reconstruction of the neck defect promoted other treatments such as radiation or chemotherapy due to the improved local situation.

## Introduction

Anaplastic thyroid carcinoma (ATC) is a very rare malignant tumor, with an incidence of only 1–2 persons per million per year. It accounts for < 2% of thyroid malignancies but has an extremely poor prognosis [1]. This group of aggressive tumors shows high invasiveness, fast growth, and poor response to systematic chemotherapy [2–6].

The skin infiltration and exophytic growth of ATC have a severe psycho-emotional impact on patients; they have difficulty fitting into society and family because of their deformity. Therefore, restoring the neck defect, and especially the esthetic look, is crucial despite the poor prognosis and short life expectancy.

The palliative resection of advanced ATC with skin invasion can lead to a significant skin defect on the neck, making direct closure impossible. In such cases, reconstructive plastic surgery is required to repair the neck defect. This reconstruction should not delay the further oncological treatment of the patients and should lead to satisfactory and fast healing.

Various coverage options by applying different flaps have been described in the literature. One of them is an internal mammary artery perforator flap (IMAP), which was first reported by Yu et al. for neck and tracheostomy reconstruction [7]. Koshima first used the term perforator flap in 1989 for paraumbilical flaps [8].

We report a case of advanced ATC, exophytically growing and with an invasion of the skin, in a patient who underwent palliative surgery and neck reconstruction with an IMAP flap.

## Case presentation

A 40-year-old Caucasian woman underwent subtotal thyroidectomy in another European country due to suspicion of a thyroid goiter. The patient had no family history of ATC. Pathological examination at that time showed thyroid goiter with signs of mesenchymal tumor. Two months later, the recurrent tumor in the neck was again resected. At that time, the malignancy was diagnosed as an ATC. Because of the histopathological diagnosis, chemoradiotherapy was administered. The patient received adjuvant chemotherapy with doxorubicin (60 mg/m^2^ every 21 days) and 36 Gy in 12 fractions postoperative external beam radiation therapy.

Unfortunately, the treatment failed to contain the tumor; within 1 month after the operation, she developed tumor progression, with fast growth of a tumor mass on the neck and was admitted to our hospital.

**Fig. 1:**
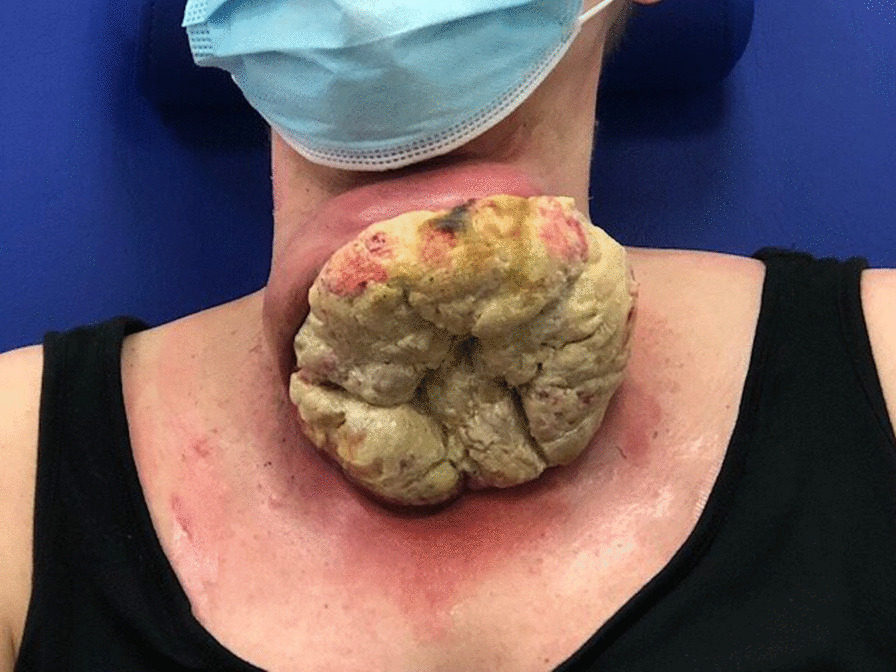
Preoperative view of the patient with a large exophytically growing tumor mass with an invasion in the surrounding skin

On admission, physical examination revealed a large exophytically growing tumor mass with an invasion in the surrounding skin and a chronic nonhealing ulcer and necrotic tissue in the tumor (Fig. 1). The patient reported significant morbidity and deterioration in her mental state. She was under high psychological stress because of the neck deformity and visible large bad-smelling tumor mass.

**Fig. 2:**
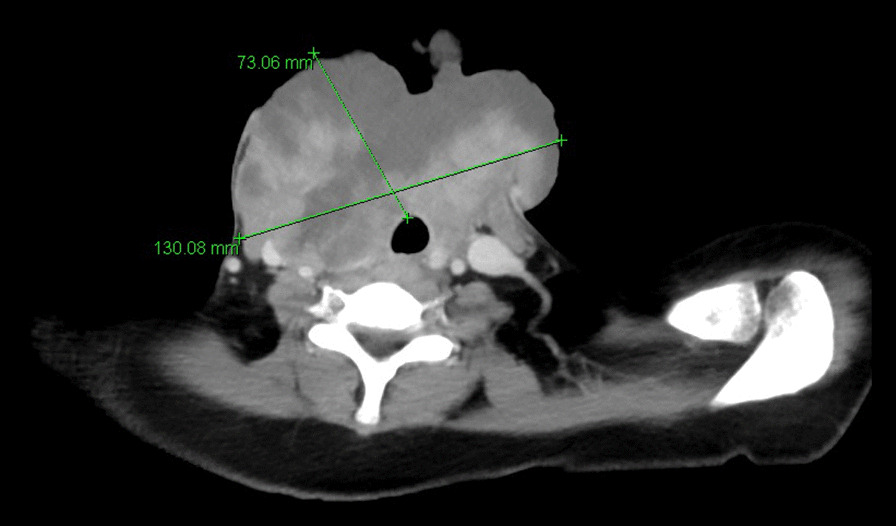
Preoperative computed tomography of the neck with a widespread (130 × 73 mm) inhomogeneous mass in the thyroid lodge

Computed tomography of the neck showed a widespread (130 × 73 mm) inhomogeneous mass in the thyroid lodge, with compression of the right jugular vein and extended to the right mediastinum to the supra-aortic vessels (Fig. 2).

**Fig. 3:**
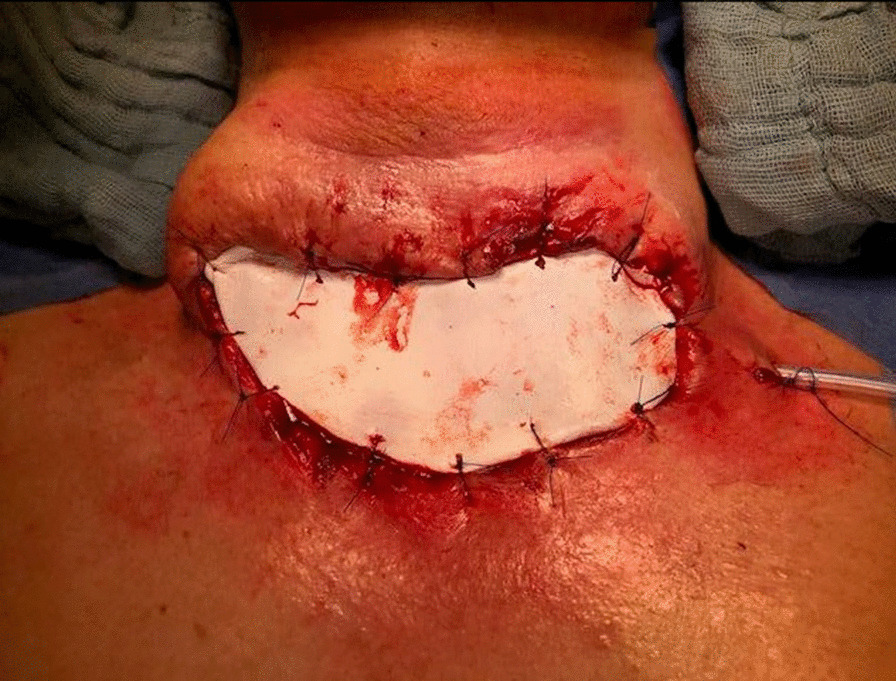
Neck defect after resection of the tumor mass temporarily covered with synthetic skin replacement

The tumor was identified as nonresectable, and palliative resection of the tumor mass was planned. The operation was carried out under general anesthesia. Due to the massive invasion of the tumor mass into surrounding tissue, the patient underwent intralesional excision, including the affected skin. This resulted in a 10 × 5 cm soft tissue defect on the neck; the skin defect was temporarily covered with synthetic skin replacement (Fig. 3). The postoperative recovery was uneventful.

**Fig. 4:**
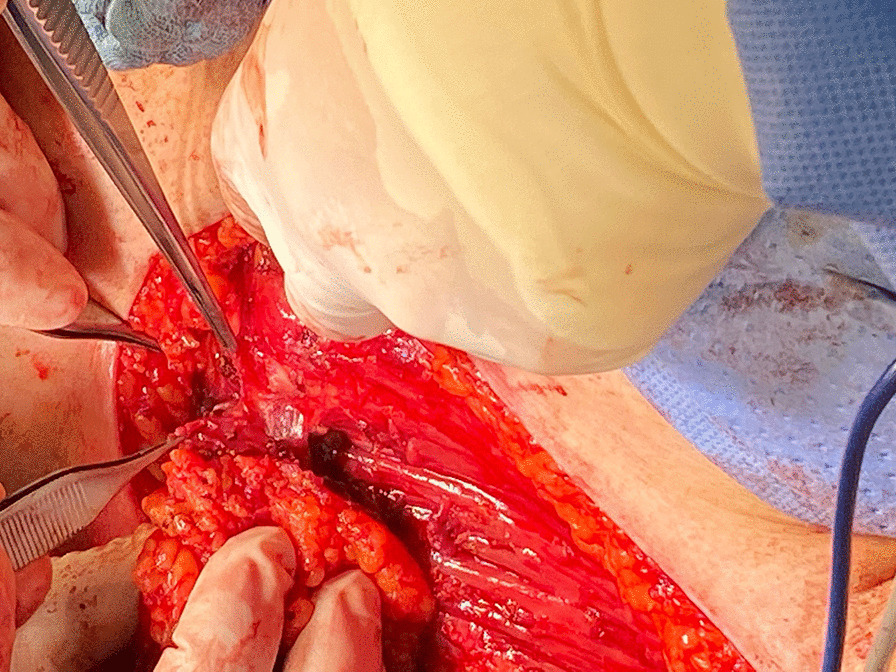
Identification of internal mammary artery perforator vessels

**Fig. 5:**
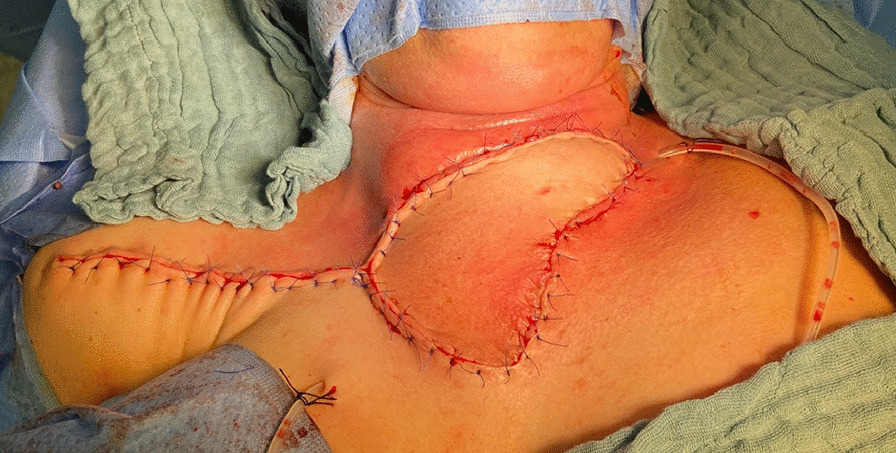
Flap inset and primary closure of donor site with two percutaneous drains placed

On the sixth postoperative day, reconstruction of the neck was performed with an IMAP flap. Initially, the internal mammary artery perforators on the right side along the sternal border were examined and marked preoperatively with a handheld Doppler. The flap based on the perforator of the second intercostal space was chosen, which was deemed the dominant vessel based on a better Doppler signal. The flap was marked so that the distal part of the flap was pulled towards the right shoulder and measured 7 × 16 cm. Skin harvesting was started from the lateral edge of the flap in a subfascial plane until the internal mammary perforator vessels were identified. The distal part of the flap extended about 2.0 cm beyond the anterior axillary fold. The perforator artery measured 2.0 mm (Fig. 4). IMAP was islanded, the narrow skin bridge between the donor site and the neck wound was divided, and the flap rotated clockwise through 120° along the vascular axis to fill the neck defect. After suturing the flap completely without tension, two percutaneous drains were placed. The donor site was closed directly without tension. The flap was constantly well-perfused (Fig. 5).

**Fig. 6:**
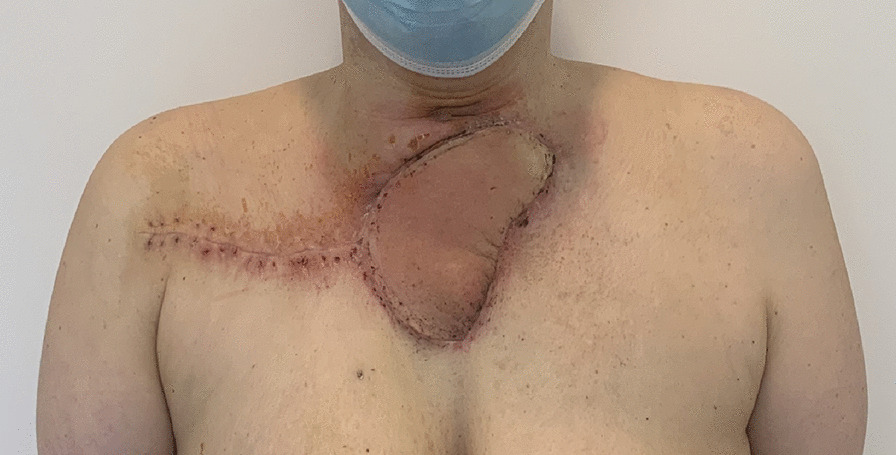
Four weeks post-surgery showing healed wound

Postoperative recovery was uneventful, and no surgical complications occurred in the postoperative period to hospital discharge on postoperative day 5. The patient was delighted with the esthetic outcome and functional results of the operation (Fig. 6). Molecular-targeted therapy with lenvatinib (10 mg) and pembrolizumab (200 mg) every month as a part of palliative therapy was initiated. Follow-up was performed every 3 months by computed (18F)2-fluoro-2-deoxy-d-glucose-positron emission tomography (FDG-PET) combined with computed tomography (CT). The patient died of disease due to a local invasion into the trachea 13 months after surgery.

## Discussion and conclusions

ATC is a rare malignancy and accounts for around 1–2% of all thyroid malignancies [1]. This carcinoma shows fast growth, strong invasiveness in the adjacent tissues, and a high rate of metastasis into the regional lymph nodes (42%) and distant organs (32%) [3, 5, 9]. Most cases (about 80%) are diagnosed in later stages and already have disease progression to the surrounding tissue and organs and distant metastasis [2, 6]. Various therapeutic modalities, including surgery, chemotherapy, and external beam radiotherapy, have been reported for these tumors [2–4, 10, 11]. Apart from these therapeutic modalities, second-line treatments, including tyrosine kinase inhibitors, have been explored in ATC patients and resulted in acceptable treatment responses [10, 12–14]. However, the ATC patients’ median overall survival is 4–8 months [2]. In many cases, the surgery for local disease management cannot be applied because of the advanced tumor, and palliative resections are not recommended because of the risk of increased morbidity [15]. Most patients die due to uncontrolled local tumor invasion or distant metastases.

Our patient’s tumor extended very fast into surrounding tissue and could not be resected completely. The patient reported a significant deterioration in her mental state, and she was under high psychological stress because of her neck deformity and the foul smell. She had difficulty fitting into family and society because of the large visible exulcerated tumor. In such cases, palliative tumor debulking can significantly improve the mental state despite the poor prognosis and short life expectancy. However, skin defects that form after tumor resection need reconstructive surgery, accelerating wound healing and facilitating the rapid application of local and systemic oncological therapy. The skin flaps should be easy to manage and have fewer complications. The primary closure of the donor site is also essential for flap selection. Therefore, we chose the IMAP island flap.

Yu et al. first described the IMAP flap for reconstructing tracheostomy and anterior neck defects. A thin and pliable fasciocutaneous flap is well suited to anterior neck defects—this flap is based on the deltopectoral axis [7]. Bakamjian et al. first described the deltopectoral flap as a pedicle flap based on the first four internal mammary perforators. These numerous perforators subsequently restrict flap mobility. Also, the deltopectoral flap requires skin grafting to the donor site defect because primary closure is difficult, which delays the healing process [16]. The IMAP flap can be islanded on a single perforator and therefore have a significantly longer arc of rotation than the deltopectoral flap [17–20]. Moreover, most authors also describe the possibility of immediate skin closure at the donor site for nearly all patients [17, 18, 18].

The perforator can be determined using a handheld Doppler device within a 1–3 cm lateral to the sternal border [20]. The flap can be raised on any perforator in the first five intercostal spaces [19]. Most flaps are based on the internal mammary perforator at the second or third intercostal space. The second has been shown to have the largest diameter perforator (1.6 ± 0.5 mm, range 0.9–2.3 mm) with an average pedicle length of 3.6 cm and perfuse the largest skin dimensions (15 × 8 cm) [18, 21, 22]. The flap is then designed around the perforator, usually obliquely or parallel to the intercostal space towards the axilla. The flap is dissected from lateral to medial and from distal to cranial. The dissection is made in a subfascial plane and proceeds until the pedicle of the internal mammary artery is identified. The flap is isolated as an islanded flap. The blood supply by only one perforator of the internal mammary vessels allows more versatility in flap design and increases the arc of rotation; the rotation can be from 90° to 180°. The flap can be brought to the neck defect through a subcutaneous tunnel, or the skin bridge between the neck and donor site can be divided. Flap sizes from 5 × 3 cm to 15 × 8 cm are reported in the literature [18]. The flap can be in a relatively short time harvest; the inset is simple [23]. The donor site, in most cases, can be primarily closed, and there are usually no side effects such as skin necrosis [18]. The IMAP flap is a reliable option that provides well-vascularized tissue ideal for reconstructing the lower neck where thin, pliable tissue is needed. Flap harvest and inset are simple, while primary closure of the donor site is a definite advantage in minimizing morbidity.

In conclusion, the palliative resection of the advanced anaplastic thyroid carcinoma and plastic reconstruction of the neck defect can promote other oncological treatments such as radiation or chemotherapy due to improved local situation. The application of the IMAP flap is a good option for neck reconstruction in such cases due to easy elevation, reliable blood supply, and minimal donor site morbidity.

## Data Availability

Data sharing is not applicable to this article as no datasets were generated or analysed during the current study.

## Abbreviations

ATC: Anaplastic thyroid carcinoma
IMAP: Internal mammary artery perforator flap
